# HCV-induced autophagosomes are generated via homotypic fusion of phagophores that mediate HCV RNA replication

**DOI:** 10.1371/journal.ppat.1006609

**Published:** 2017-09-19

**Authors:** Linya Wang, Ja Yeon Kim, Helene Minyi Liu, Michael M. C. Lai, Jing-hsiung James Ou

**Affiliations:** 1 Department of Molecular Microbiology and Immunology, University of Southern California, Keck School of Medicine, Los Angeles, California, United States of America; 2 Institute of Biochemistry and Molecular Biology, College of Medicine, National Taiwan University, Taipei, Taiwan; 3 Research Center for Emerging Viruses, China Medical University Hospital and China Medical University, Taichung, Taiwan; University of California, San Diego, UNITED STATES

## Abstract

Hepatitis C virus (HCV) induces autophagy to promote its replication, including its RNA replication, which can take place on double-membrane vesicles known as autophagosomes. However, how HCV induces the biogenesis of autophagosomes and how HCV RNA replication complex may be assembled on autophagosomes were largely unknown. During autophagy, crescent membrane structures known as phagophores first appear in the cytoplasm, which then progress to become autophagosomes. By conducting electron microscopy and *in vitro* membrane fusion assay, we found that phagophores induced by HCV underwent homotypic fusion to generate autophagosomes in a process dependent on the SNARE protein syntaxin 7 (STX7). Further analyses by live-cell imaging and fluorescence microscopy indicated that HCV-induced phagophores originated from the endoplasmic reticulum (ER). Interestingly, comparing with autophagy induced by nutrient starvation, the progression of phagophores to autophagosomes induced by HCV took significantly longer time, indicating fundamental differences in the biogenesis of autophagosomes induced by these two different stimuli. As the knockdown of STX7 to inhibit the formation of autophagosomes did not affect HCV RNA replication, and purified phagophores could mediate HCV RNA replication, the assembly of the HCV RNA replication complex on autophagosomes apparently took place during the formative stage of phagophores. These findings provided important information for understanding how HCV controlled and modified this important cellular pathway for its own replication.

## Author summary

Autophagy is a catabolic process that is important for maintaining cellular homeostasis. During autophagy, crescent membrane structures known as phagophores first appear in the cytoplasm, which then expand to form enclosed double-membrane vesicles known as autophagosomes. It has been shown that hepatitis C virus (HCV) induces autophagy and uses autophagosomal membranes for its RNA replication. In this report, we studied the biogenesis pathway of HCV-induced autophagosomes and demonstrated that phagophores induced by HCV originated from the endoplasmic reticulum and undergo homotypic fusion to generate autophagosomes, and that the HCV RNA replication complex is assembled on phagophores prior to the formation of autophagosomes. These findings provided important information for understanding how an RNA virus controls this important cellular pathway for its replication.

## Introduction

Autophagy is a catabolic process that is important for maintaining cellular homeostasis. It begins with the formation of membrane crescents termed phagophores or isolation membranes in the cytosol. The membranes of phagophores will subsequently expand to sequester part of the cytoplasm, resulting in the formation of enclosed double-membrane vesicles known as autophagosomes. Autophagosomes mature by fusing with lysosomes to form autolysosomes, in which the cargos of autophagosomes are digested by lysosomal enzymes [1]. The phagophore assembly site (PAS), also known as the pre-autophagosomal structure, may be located on the endoplasmic reticulum (ER) or other intracellular membranes [2]. In the canonical autophagic pathway, the class III phosphatidylinositol-3-kinase (PI3KC3) mediates the production of phosphatidylinositol-3-phosphate (PI3P), which then recruits PI3P-binding proteins such as DFCP1 or WIPI to the PAS to form omegasomes [3]. This is followed by the recruitment of autophagy-related proteins ATG5 and ATG12, which are covalently linked, and ATG16, leading to the formation of phagophores. LC3 is the microtubule-associated protein light chain 3. Its non-lipidated form (i.e., LC3-I) is located in the cytosol. However, during autophagy, LC3 becomes lipidated (i.e., LC3-II) and eventually replaces the ATG5-ATG12-ATG16 complex on growing phagophores. LC3-II remains associated with autophagosomes after they are formed [4, 5]. ATG5, ATG12 or ATG16 is thus often used as the marker for phagophores and LC3-II is often used as the marker for autophagosomes [6].

Autophagy occurs in cells at a basal level and can be induced by stresses such as nutrient starvation. It can also be induced by microbial infections for the removal of intracellular microbial pathogens. However, many microbial pathogens including viruses have also developed mechanisms to subvert this intracellular anti-microbial pathway and even use this pathway to enhance their own replications. In recent years, many reports have been published to show that hepatitis C virus (HCV) could induce autophagy to support its own replication [7–11]. HCV is a hepatotropic virus that can cause severe liver diseases including cirrhosis and hepatocellular carcinoma. It has a 9.6-Kb positive-stranded RNA genome that encodes a polyprotein with a length of slightly more than 3,000 amino acids. After its synthesis, the HCV polyprotein is proteolytically cleaved into structural and nonstructural proteins by cellular and viral proteases [12]. The nonstructural proteins NS3, NS4A, NS4B, NS5A and NS5B are required for viral RNA replication [13], which can take place on autophagosomal membranes [14].

Although it has been very well documented that HCV could induce autophagy to enhance its own replication [15], the biogenesis pathway of autophagosomes induced by HCV remains unclear. Phagophores were thought to extend their membranes to form the enclosed autophagosomes, but more recent studies indicated that they could also undergo homotypic fusion to generate autophagosomes in a process dependent on soluble N-ethylmaleimide-sensitive factor activating protein receptor (SNARE) proteins [16], which play important roles in mediating the fusion of vesicular membranes in cells [17]. Whether and how phagophores are involved in the biogenesis of autophagosomes induced by HCV are largely unknown.

In this report, we conducted electron microscopy to examine Huh7 hepatoma cells that harbored an HCV subgenomic RNA replicon and identified crescent membrane structures that resembled phagophores, which appeared to be able to undergo homotypic fusion to form autophagosomes. By conducting an *in vitro* membrane fusion assay, we demonstrated that ATG5-positive phagophores induced by HCV could indeed undergo homotypic fusion to form autophagosomes. We also discovered that, comparing with autophagy induced by nutrient starvation, the progression of phagophores to autophagosomes induced by HCV was prolonged, and that the HCV RNA replication complex was assembled on phagophores prior to the formation of autophagosomes.

## Results

### Homotypic fusion of phagophores for the assembly of autophagosomes

**Fig 1:**
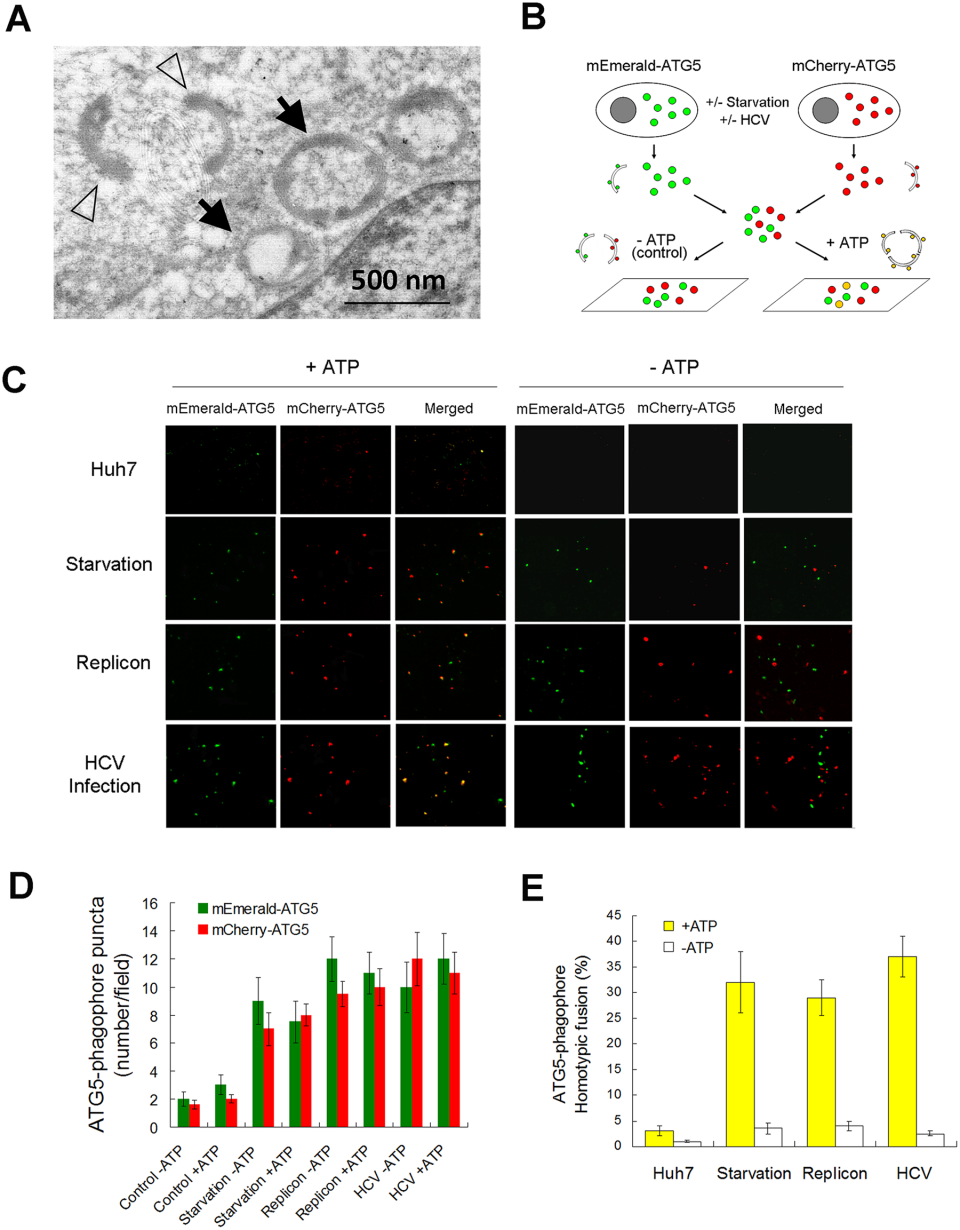
Analysis of homotypic fusion of phagophores. (A) Electron microscopy of the HCV replicon cell. Open arrowheads and black arrows denote phagophore-like membrane structures and autophagosomes, respectively. The large black arrow denotes an autophagosome that appeared to be assembled via the fusion of several phagophores. N, nucleus. (B) Illustration of the *in vitro* homotypic fusion assay of phagophores. See text for details. (C) Analysis of homotypic fusion of phagophores *in vitro*. Huh7 cells were transfected with the expression plasmid for mEmerald-ATG5 (green) or mCherry-ATG5 (red). Two days after the DNA transfection, cells with or without nutrient starvation for one hour were lysed for the phagophore fusion assay in the presence (+ATP) or absence (-ATP) of ATP. Nutrient starvation was conducted by incubating cells in Hanks balanced salt solution (HBSS). HCV replicon cells were similarly transfected with the ATG5-expressing plasmids and harvested 2 days after transfection (Replicon) for the membrane fusion assay. For HCV infection studies, Huh7.5 cells were transfected with the ATG5-expressing plasmid and, one day after transfection, infected with HCV (m.o.i. = 1) for 1 day and then lysed for the *in vitro* phagophore fusion assay. The fusion results were analyzed by fluorescence microscopy. (D) Quantification of mEmerald-ATG5 and mCherry-ATG5 puncta shown in (C). (E) Quantification of the homotypic fusion efficiency of ATG5 puncta shown in (C).

To understand how HCV induces the biogenesis of autophagosomes, we performed electron microscopy on HCV replicon cells, which contained the self-replicating HCV subgenomic RNA that expressed the HCV nonstructural proteins NS3-NS5B [18]. As shown in Fig 1A, in agreement with our previous reports [14], autophagosomes approximately 400–500 nm in diameters could be detected in replicon cells. Two crescent membranous structures that resembled phagophores were also detected. Interestingly, one of the autophagosomes observed appeared to be assembled by three phagophore-like structures. This result raised the possibility that autophagosomes induced by HCV might be generated via the homotypic fusion of phagophores. To test this possibility, we expressed the mEmerald-ATG5 fusion protein and the mCherry-ATG5 fusion protein separately in Huh7 cells by transient transfection to label phagophores. Cells were then lysed with a hypotonic buffer and cell lysates containing either the mEmerald-ATG5-labeled phagophores or the mCherry-ATG5-labeled phagophores were then mixed for the *in vitro* membrane fusion assay. If phagophores could undergo homotypic fusion, then mEmerald-ATG5-labeled and mCherry-ATG5 labeled phagophores would be expected to merge to generate yellow puncta when they were visualized under a fluorescence microscope (Fig 1B). We first performed a positive control experiment using cells that were nutrient-starved, which would induce autophagy. As shown in Fig 1C and 1D, only a few mEmerald-ATG5 and mCherry-ATG5 puncta (i.e., phagophores) could be detected in control Huh7 cell lysates. However, their levels were significantly increased if cells were nutrient-starved. In the presence of ATP, approximately 30% of phagophores from nutrient-starved cells could undergo homotypic fusion, as evidenced by the merging of mEmerald-ATG5 and mCherry-ATG5 puncta (Fig 1C and 1E), in agreement with the previous report [16]. This homotypic fusion, which is ATP-dependent, was not observed in the absence of ATP, which served as the negative control. Few phagophores from control Huh7 cells could undergo homotypic fusion even in the presence of ATP (Fig 1E), presumably due to the low concentration of phagophores. We then examined the HCV replicon cells that were also transfected with the expression plasmids of mEmerald-ATG5 and mCherry-ATG5. As shown in Fig 1C and 1D, HCV replicon cells also contained an increased level of phagophores. The same as nutrient-starved cells, approximately 30% of phagophores isolated from HCV replicon cells could undergo homotypic fusion in the presence of ATP but not in the absence of it (Fig 1C and 1E). Similar results were observed with Huh7.5 cells infected by HCV (Fig 1C and 1E).

### Essential role of STX7 in the homotypic fusion of phagophores induced by HCV

**Fig 2:**
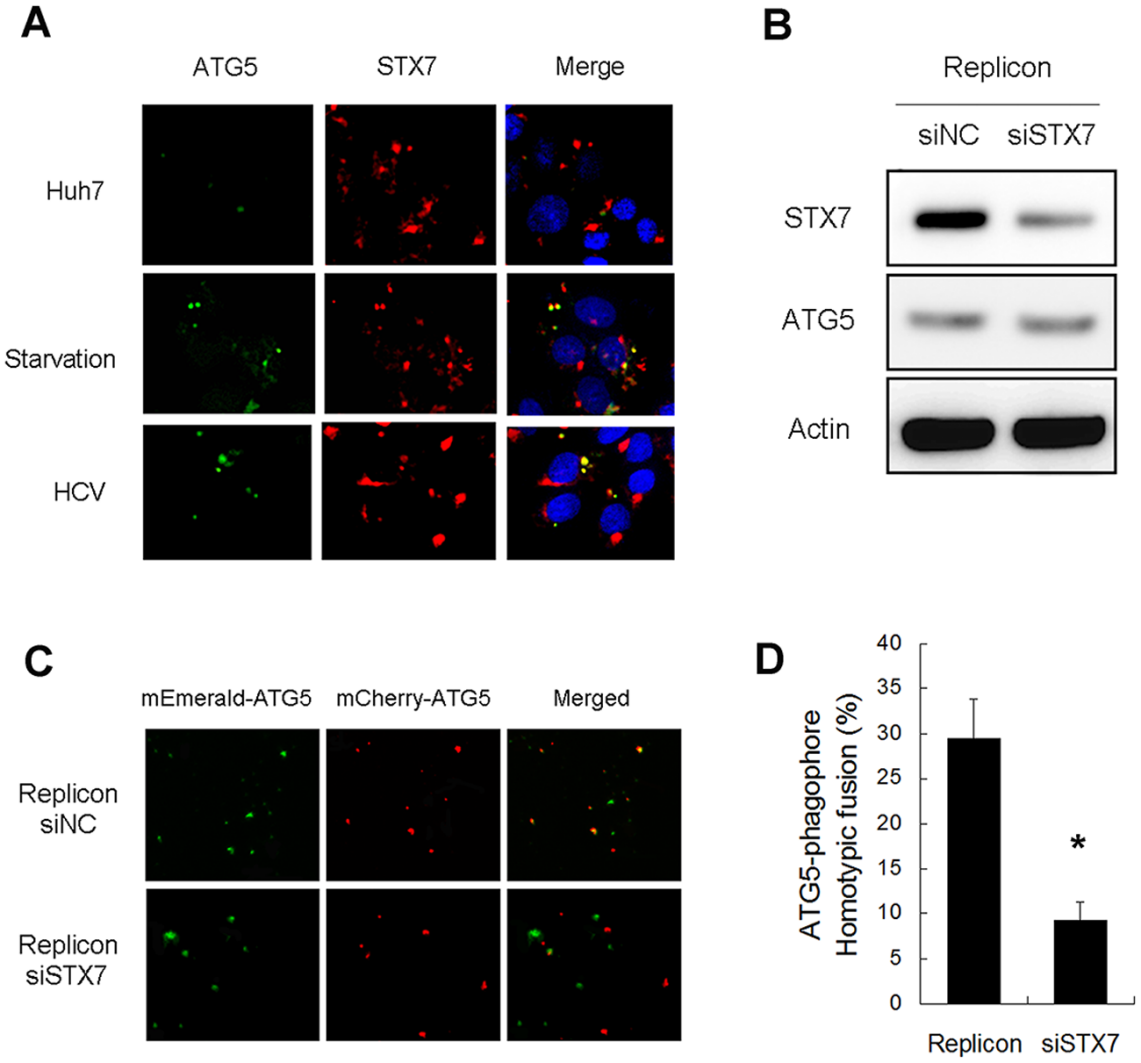
Knockdown of STX7 inhibits homotypic fusion. (A) Colocalization of ATG5 and STX7. Cells were transfected with mEmerald-ATG5 (green) for 2 days, followed by nutrient starvation for one hour or HCV infection for one day, and then stained with anti-STX7 antibody (red) for fluorescence microscopy. DAPI (blue) was used to stain nuclei. (B) Western-blot analysis of STX7 and ATG5 in replicon cells transfected with the control siRNA (siNC) or siSTX7 for two days. (C) *In vitro* fusion assay of phagophores isolated from replicon cells with or without STX knockdown. HCV replicon cells were transfected with the expression plasmid for mEmerald-ATG5 or mCherry-ATG5 for one day and then further transfected with siNC or siSTX7 for two days. Cells were then lysed for the *in vitro* membrane fusion assay. (D) Quantification of the homotypic fusion efficiency of phagophores shown in (C). *, p < 0.005.

The SNARE protein STX7 plays important roles in mediating the fusion of intracellular membrane vesicles. Our recent studies indicated that STX7 was associated with autophagosomes [19]. To determine whether STX7 also mediates the homotypic fusion of phagophores, we first examined whether STX7 was associated with phagophores. Huh7 cells were transfected with the mEmerald-ATG5-expressing plasmid and stained with the anti-STX7 antibody. As shown in Fig 2A, few mEmerald-ATG5 puncta could be detected in Huh7 cells. However, in agreement with the results shown in Fig 1C, the number of mEmerald-ATG5 puncta in Huh7 cells was increased by nutrient starvation, indicative of the induction of autophagy. Most of the mEmerald-ATG5 puncta were also positive for STX7. The same results were also observed in HCV-infected cells. These results indicated that STX7 was associated with phagophores induced by nutrient starvation and HCV.

To determine whether STX7 could mediate the homotypic fusion of the phagophores induced by HCV, we expressed mEmerald-ATG5 and mCherry-ATG5 separately in HCV replicon cells followed by the suppression of STX7 using the siRNA (siSTX7). The knockdown of STX7 had no effect on the expression of ATG5 (Fig 2B). We then repeated the *in vitro* membrane fusion assay. As shown in Fig 2C, although the knockdown of STX7 had no apparent effect on the level of ATG5 puncta in HCV replicon cells, it reduced the percentage of homotypically fused phagophores from approximately 30% to slightly less than 10% (Fig 2D). These results indicated that, although STX7 was not required for the production of phagophores, it was required for their efficient homotypic fusion.

### Requirement of STX7 for the formation of autophagosomes induced by HCV

**Fig 3:**
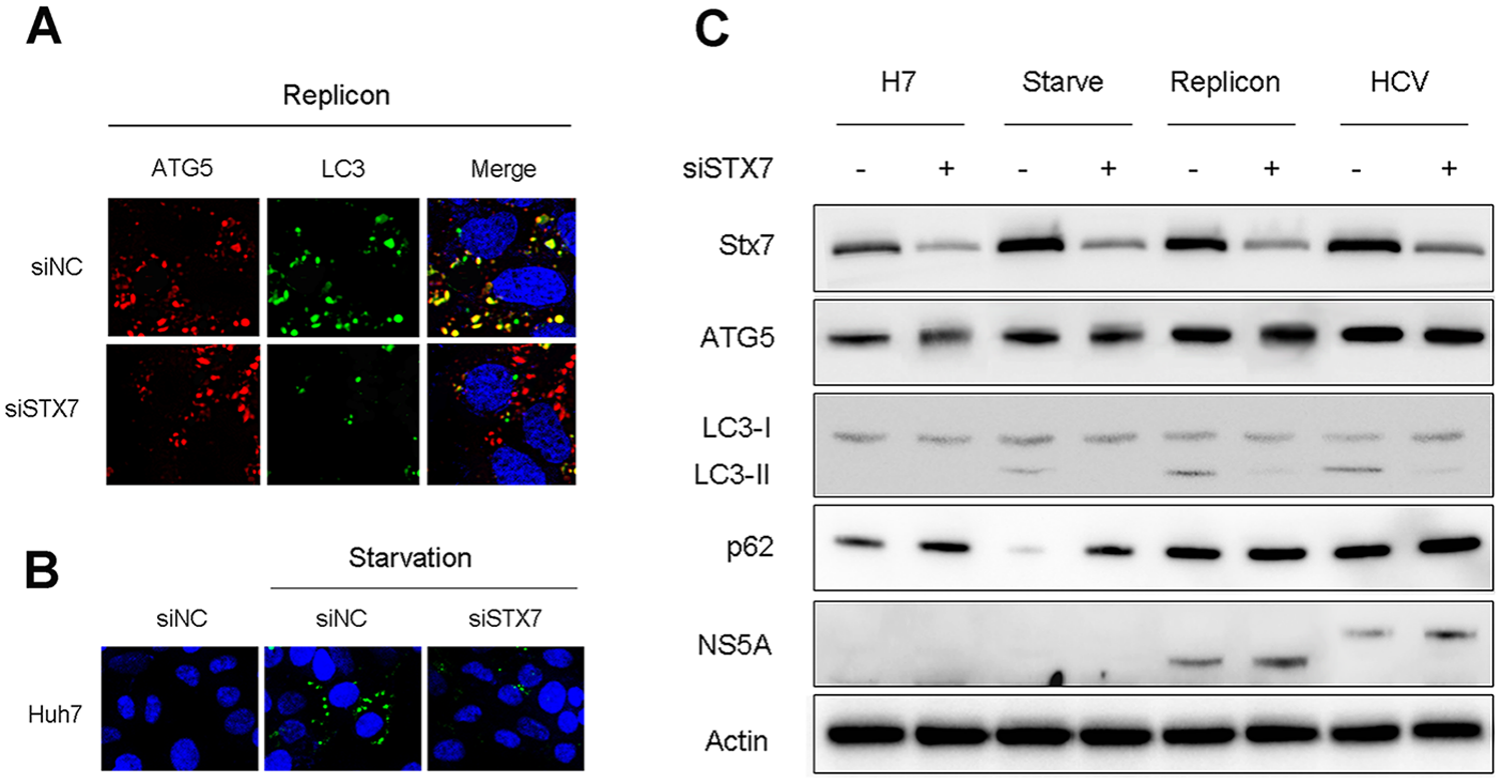
Requirement of STX7 for the formation of autophagosomes induced by HCV. (A) Loss of autophagosomes but not phagophores in HCV replicon cells after STX7 knockdown. HCV replicon cells stably expressing the GFP-LC3 fusion protein were transfected with mCherry-ATG5 for one day and then further transfected with the control siRNA (siNC) or siSTX7 for two days. Cells were then fixed for fluorescence microscopy. DAPI (blue) stained for nuclei. (B) Loss of autophagosomes in nutrient-starved cells after STX7 knockdown. Huh7 cells stably expressing GFP-LC3 were transfected with control siRNA (siNC) or siSTX7 and incubated in HBSS solution for one hour for nutrient starvation. (C) Western-blot analysis for autophagic proteins in cells with and without STX7 knockdown. Huh7 and replicon cells were transfected with the control siRNA or siSTX7 for two days, and Huh7 cells were nutrient-starved for one hour before being harvested for western-blot analysis. For HCV infection, cells were transfected with siSTX7 for one day and then infected with HCV (m.o.i. = 1) for another day before western-blot analysis.

To determine whether the homotypic fusion of phagophores is required for the formation of autophagosomes induced by HCV, we transfected HCV replicon cells that stably expressed the GFP-LC3 fusion protein with the mCherry-ATG5-expressing plasmid and then knocked down the expression of STX7 using the siRNA. If the homotypic fusion of phagophores is required for the formation of autophagosomes, then its inhibition should reduce the level of autophagosomes induced by HCV. As shown in Fig 3A, the suppression of STX7 expression did not affect the level of mCherry-ATG5 puncta (i.e., phagophores), but it significantly reduced the level of GFP-LC3 puncta (i.e., autophagosomes). This result demonstrated that STX7 was not essential for the formation of phagophores but it was required for the formation of autophagosomes induced by HCV. The knockdown of STX7 also suppressed the formation of autophagosomes in nutrient-starved cells (Fig 3B).

To further confirm the fluorescence imaging results shown in Fig 3A and 3B, we also performed the western-blot analysis. As shown in Fig 3C, the suppression of STX7 expression with siSTX7 had only a marginal effect, if any, on the levels of ATG5 in control Huh7 cells, nutrient-starved Huh7 cells, HCV replicon cells and HCV-infected cells. It also had no apparent effect on LC3 in control Huh7 cells, which had a low level of lipidated LC3 (i.e., LC3-II), a marker of autophagosomes. However, STX7 knockdown suppressed the induction of LC3-II in nutrient-starved Huh7 cells, HCV replicon cells, and HCV-infected cells. These results confirmed that STX7 was important for the formation of autophagosomes induced by nutrient starvation or HCV. The analysis of p62, a protein degraded by autophagy, revealed that the suppression of STX7 marginally increased the p62 level in control Huh7 cells, likely due to the inhibition of the basal autophagy, and prevented the loss of p62 induced by nutrient starvation. These results indicated the importance of STX7 in the completion of the autophagic flux and the autophagic protein degradation. Comparing with control Huh7 cells, HCV replicon cells and Huh7 cells infected by HCV for one day had an increased level of p62. This result was consistent with our previous finding that HCV induced the expression of RUBICON in replicon cells and in the early time point of infection to suppress the fusion between autophagosomes and lysosomes [18]. The knockdown of STX7 did not further increase the level of p62 in replicon cells and HCV-infected cells, presumably because the autophagic flux and the autophagic protein degradation had already been suppressed by HCV in replicon cells and in infected cells at this time point. Note that HCV NS5A of replicon cells migrated faster in the gel than that of HCV-infected cells. This was due to the adaptive mutation in replicon NS5A, which prevented its hyperphosphorylation [20, 21], and likely also due to the fact that HCV replicon RNA was derived from HCV genotype 1b whereas HCV JFH1, which we used for the infection studies, was a genotype 2a virus. The fluorescence microscopy results (Fig 3A and 3B) and the western-blot results (Fig 3C) together demonstrated that STX7 was essential for the formation of autophagosomes induced by HCV as well as by nutrient starvation.

VAMP7 is another SNARE protein that had previously been shown to be important for the homotypic fusion of phagophores [16]. We therefore also tested the possible effect of VAMP7 knockdown on the formation of phagophores and autophagosomes in nutrient-starved cells and HCV replicon cells. Our results indicated that VAMP7 knockdown had no effect on phagophores in nutrient-starved cells and HCV replicon cells, but it suppressed the formation of autophagosomes in both cases (S1A Fig). We also conducted the western-blot analysis. As shown in S1B Fig, VAMP7 knockdown had no effect on STX7 and ATG5, but it reduced LC3-II level in nutrient-starved cells and replicon cells. It also restored the p62 level in nutrient-starved cells. These results were consistent with STX7 knockdown and further confirmed that the homotypic fusion of phagophores was important for the formation of autophagosomes induced by nutrient starvation and HCV.

### Generation of phagophores from the endoplasmic reticulum (ER) and their progression into autophagosomes

**Fig 4:**
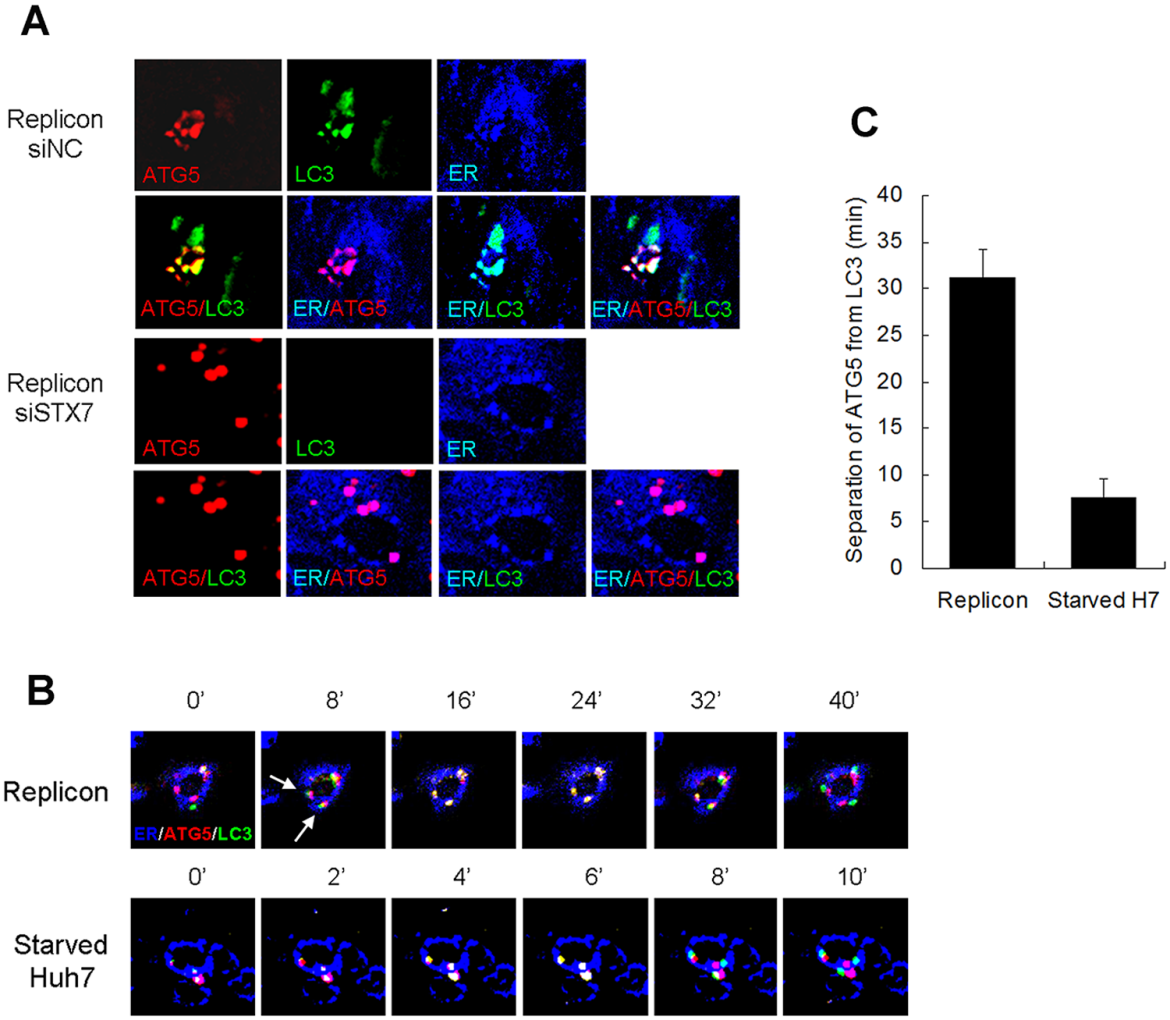
Analysis of the progression of phagophores into autophagosomes in HCV replicon cells. (A) Colocalization analysis of ATG5 (red), LC3 (green) and ER (blue) in HCV replicon cells. HCV replicon cells stably expressing GFP-LC3 were transfected with the mCherry-ATG5-expressing plasmid for one day and then with the control siRNA or siSTX7 for two days. Cells were then stained with ER Tracker Blue. (B) Live cell imaging analysis. HCV replicon cells (upper panels) and nutrient-starved Huh7 cells (lower panels) that stably expressed LC3-GFP were transfected with the mCherry-ATG5-expressing plasmid for 2 days and then stained with ER Tracker Blue for 40 minutes before imaging. The HCV replicon cells were observed for 40 mins with images taken once every 8 minutes. Huh7 cells were starved for 1 hour and observed for 10 minutes with images taken once every 2 minutes. Noe that, in HCV replicon cells, the two mCheery-ATG5 puncta denoted by arrows were associated with the ER and became entirely GFP-LC3-positive (i.e., yellow in color) at the 16-minute time point. The GFP-LC3 puncta did not separate from the mCherry-ATG5 puncta until at the 40-minute time point. In contrast, all three mCherry-ATG5 puncta in nutrient-starved Huh7 cells became GFP-LC3-positive at the 2-4-minute time points and were separated from GFP-LC3 puncta at the 8-10-minute time points. (C) Quantification of the average time length required for LC3 puncta to separate from ATG5 puncta. More than 10 puncta were observed for each cell type.

Phagophores may be derived from the ER or other cellular membranes [22]. To investigate whether phagophores induced by HCV might be derived from the ER, we transfected HCV replicon cells that stably expressed GFP-LC3 with the mCherry-ATG5-expressing plasmid and stained the ER using ER Tracker Blue. As shown in Fig 4A, mCherry-ATG5 colocalized with the ER in HCV replicon cells, providing the evidence that phagophores induced by HCV might be originated from ER membranes. Most of the mCherry-ATG5 puncta were also positive for GFP-LC3, indicating the progression of phagophores into autophagosomes. The same results were obtained when endogenous ATG5 and LC3 were analyzed by immunofluorescence staining. As shown in S2 Fig, endogenous ATG5 and LC3 colocalized with each other and with the ER in replicon cells and HCV-infected cells. To further confirm that phagophores were indeed associated with the ER, we also analyzed the subcellular localization of endogenous ATG16, another marker of phagophore. Huh7 cells or HCV replicon cells were transfected with the mCherry-ATG5-expressing plasmid and then stained with ER Tracker blue and the anti-ATG16 antibody. As shown in S3 Fig, few ATG16 and mCherry-ATG5 puncta could be detected in control Huh7 cells. However, their numbers were significantly increased in replicon cells, and all of the ATG16 puncta colocalized with mCherry-ATG5 puncta on the ER. The analysis ATG16 in HCV-infected cells generated the same result. These results provided another line of evidence that HCV could induce phagophores, which likely originated from the ER. In agreement with the results shown in Fig 3, the suppression of STX7 expression with siSTX7 had no effect on the association of mCherry-ATG5 puncta with the ER, but it reduced the GFP-LC3 puncta to an almost undetectable level (Fig 3A). This result further demonstrated that the formation of phagophores on the ER was independent of STX7 in HCV replicon cells. Similarly, the suppression of STX7 expression had no effect on the level of ATG5 puncta, nor their association with the ER, in Huh7 cells that were nutrient-starved or infected by HCV (S4 Fig).

To confirm that mCherry-ATG5-labeled phagophores could indeed progress to become autophagosomes, we conducted the live-cell imaging. As shown in Fig 4B, mCherry-ATG5 puncta (red) first appeared on the ER, which was stained by the ER Tracker Blue. The mCherry-ATG5 puncta then progressed to become GFP-LC3-positive (yellow) followed by the separation of GFP-LC3 puncta (green) from the mCherry-ATG5 puncta and the ER. Surprisingly, the time period starting from the association of GFP-LC3 with mCherry-ATG5 puncta to their separation was roughly about 30 minutes (Fig 4B and 4C, and S1 Video), significantly longer than what was previously reported for the progression of phagophores to autophagosomes in nutrient-starved cells, which was less than 10 minutes [23]. Indeed, as a control, we also conducted the live-cell imaging on nutrient-starved Huh7 cells. As shown in Fig 4B, lower panels, and S2 Video, the time needed for the separation of GFP-LC3 from mCherry-ATG5 puncta was less than 10 minutes (Fig 4C), in agreement with the previous report [23]. These results indicated that the progression of phagophores to autophagosomes was prolonged in HCV replicon cells.

### Phagophores as the site for HCV RNA replication

**Fig 5:**
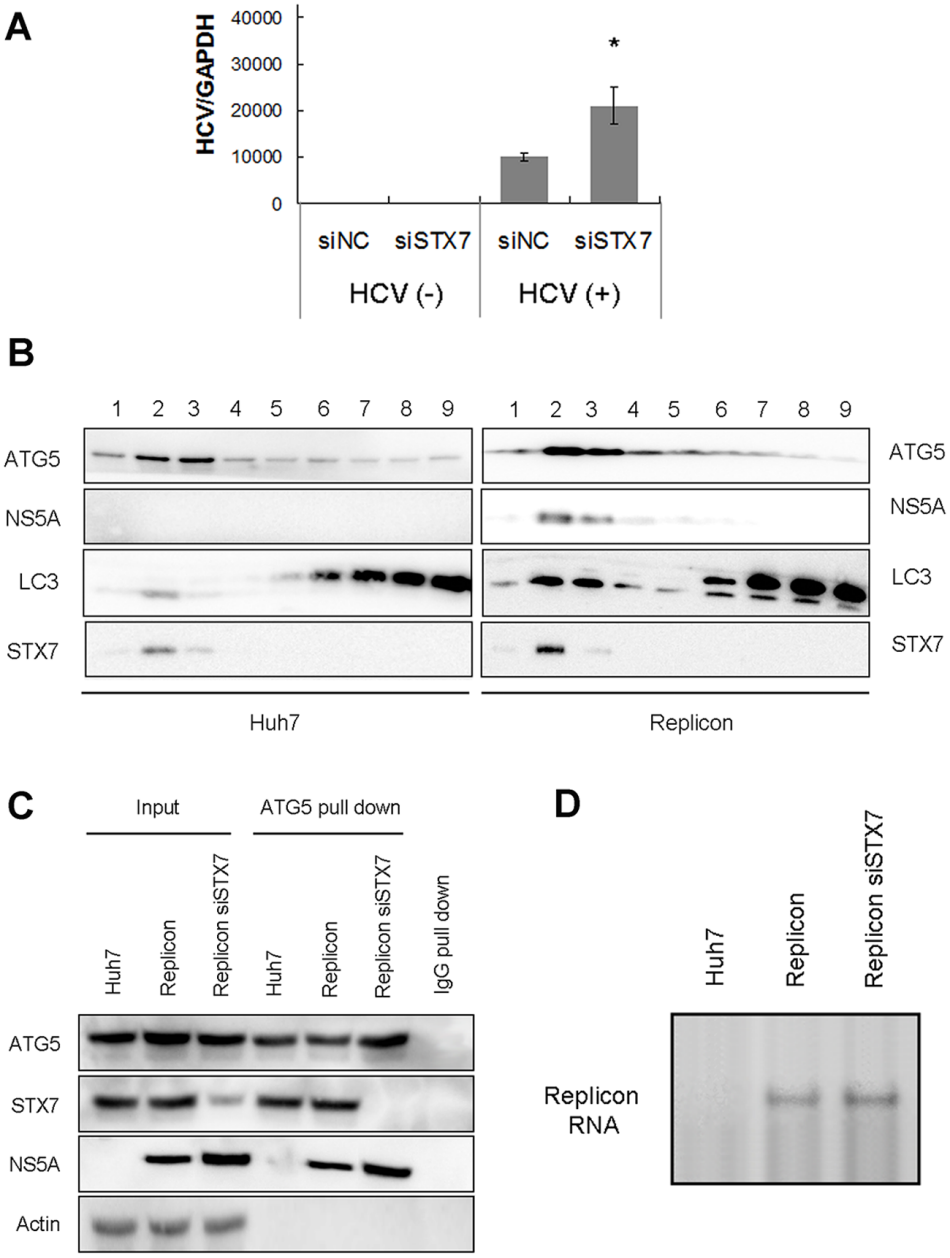
Phagophores mediated HCV RNA replication. (A) Effect of STX7 knockdown on HCV RNA. Huh7.5 cells were transfected with the control siRNA (siNC) or siSTX7 for one day and then infected with HCV for one more day. Cells were then lysed for quantification of HCV RNA using qRT-PCR. *, p < 0.05 (B) Membrane flotation analysis of control Huh7 cells and HCV replicon cells. Cells were lysed with a hypotonic buffer and membranes were separated from the cytosol by membrane flotation using a discontinuous sucrose gradient. Individual fractions were collected for western-blot analysis. (C) Affinity purification of phagophores. Cellular membranes enriched in fractions 2 and 3 of the sucrose gradient were pooled, and phagophores were affinity purified using the anti-ATG5 antibody. A control IgG was also used to purify phagophores from replicon cells to serve as a negative control. Total cell lysates were used as the input control. (D) Affinity purified phagophores were used for the *in vitro* HCV RNA replication assay.

Our previous studies indicated that autophagosomes could serve as the sites for HCV RNA replication [14, 24]. For that reason, we also determined whether phagophores could support HCV RNA replication. To test this possibility, we suppressed the expression of STX7 using a scrambled siRNA or siSTX7 followed by HCV infection for one day. Cells were then harvested and analyzed for HCV RNA replication by quantitative RT-PCR (qRT-PCR). As shown in Fig 5A, the suppression of STX7 expression, which prevented the homotypic fusion of phagophores and the generation of autophagosomes, did not decrease, but rather slightly increased the HCV RNA level. This result was consistent with the western-blot result shown in Fig 3C, which indicated that the STX7 knockdown did not decrease, but instead appeared to marginally increase the HCV NS5A protein level. To further determine whether HCV could indeed replicate on phagophores, we developed a procedure to purify phagophores from HCV replicon cells. This procedure included first, the isolation of membranes from HCV replicon cells by membrane flotation using a discontinuous sucrose gradient, and second, the affinity purification of phagophores using the anti-ATG5 antibody. As shown in Fig 5B, the membrane flotation using the sucrose gradient led to the separation of membrane-associated LC3-II from its cytosolic form LC3-I. ATG5, STX7 and the HCV NS5A protein were also enriched in the membrane fractions. Membranes enriched in fractions 2 and 3 were then incubated with the anti-ATG5 antibody followed by incubation with protein G-conjugated magnetic beads for the affinity purification of ATG5-positive membranes (i.e., phagophores). As shown in Fig 5C, the purified phagophores contained ATG5 and STX7. This result was consistent with the result shown in Fig 2A, which indicated that STX7 was associated with phagophores. The suppression of STX7 expression with siSTX7 did not affect the isolation of phagophores, again in agreement with the Fig 3A results, which indicated that STX7 was not essential for the formation of phagophores. HCV NS5A was also co-purified with phagophores, indicating the possible association of the HCV RNA replication complex with these membrane structures. In contrast, the cytosolic protein actin, which was not expected to be associated with phagophores, was not detected. ATG5, STX7 and NS5A were not detected when the control IgG was used for the affinity purification of phagophores. The purified phagophores were then tested for their abilities to direct HCV RNA replication *in vitro*. As shown in Fig 5D, the purified phagophores could indeed mediate HCV RNA replication and this replication was not inhibited by STX7 knockdown. The replicated HCV RNA was not detected when the control IgG was used for the affinity purification of phagophores (S5 Fig).

## Discussion

Autophagosomes may be derived from multiple membrane sources, including the ER [3, 22, 25], the outer membrane of mitochondria [26], the Golgi [27], early endosomes [28], plasma membranes [29], and vesicles budding from ER and Golgi [30, 31]. The ER-mitochondria junction [32] and the ER-Golgi intermediate compartment (ERGIC) [2] had also been found to participate in autophagosome biogenesis. It is possible that in different cell types, phagophores may originate from different subcellular compartments, and even within the same cell, their sites of origin may change in response to different external stimuli. As we found that phagophores induced by HCV were associated with the ER and could progress to become autophagosomes, autophagosomes induced by HCV most likely originated from ER membranes (Fig 4).

When the phagophore originates from the ER, it forms a distinct membrane structure, which elongates while being encircled by the associated ER. The edges of the phagophore are eventually sealed to form the autophagosome [22, 33]. However, it was recently reported that phagophores could also undergo homotypic fusion [16, 34]. Our results indicated that phagophores induced by HCV could also undergo homotypic fusion (Fig 1), and this homotypic fusion was dependent on the SNARE protein STX7 (Fig 2). As STX7 colocalized with phagophores induced by HCV (Fig 2A), it is conceivable that STX7 interacts with its SNARE protein partners such as VAMP7 to mediate the fusion of phagophores in HCV-infected cells (S1 Fig). This homotypic fusion of phagophores apparently was essential for the generation of autophagosomes, as its inhibition via the suppression of STX7 or VAMP7 expression abolished the production of autophagosomes without affecting phagophores (Fig 3).

Our previous studies indicated that HCV could use autophagosomes as the sites for its RNA replication [14, 24]. The unique role of STX7 in the formation of autophagosomes but not in the formation of phagophores allowed us to examine whether the HCV RNA replication complex was assembled on autophagosomes before or after their formation. Our HCV RNA replication studies using HCV replicon cells treated with siSTX7, which inhibited the formation of autophagosomes, and our cell-free HCV RNA replication assay using purified phagophores clearly demonstrated that HCV RNA replication could take place on phagophores (Fig 5). Recently, it was demonstrated that the HCV NS5A protein could transiently colocalize with omegasomes [35], and HCV NS5B RNA polymerase could transiently interact with ATG5 [36]. Thus, it is tempting to speculate that the assembly of the HCV RNA replication complex is an early event in the biogenesis of autophagosomes, and by the time when phagophores are formed, the assembly of the HCV RNA replication complex has been completed. This replication complex then remains associated with autophagosomes to continue to mediate HCV RNA replication. More recently, it was reported that the suppression of ATG12, which is required for the formation of phagophores, led to the suppression of HCV RNA replication [37]. Their results suggested an essential role of phagophores in HCV RNA replication and, together with our results, would argue that the HCV RNA replication complex was assembled initially on phagophores. It should be noted that the HCV RNA replication had also been reported to be associated with double membrane vesicles (DMVs) that were devoid of LC3 [38]. These DMVs were derived from the ER. The relationship between phagophores and DMVs is unclear and the possibility that phagophores may also be the predecessors of DMVs cannot be ruled out.

ATG5 is located on phagophores and dissociates from these membranes upon the formation of autophagosomes. We were able to identify early phagophores (ATG5-positive, LC3-negative), late phagophores (ATG5-positive, LC3-positive) and autophagosomes (ATG5-negative, LC3-positive) in HCV replicon cells. We also discovered that the kinetics of autophagic flux was different between those induced by nutrient starvation and HCV. The transition from late phagophores to autophagosomes induced by nutrient starvation was completed within 10 minutes whereas that induced by HCV took approximately 30 minutes. How HCV prolonged the transitioning from late phagophores to autophagosomes is unclear. This may be due to its use of distinct molecular pathways to induce autophagy such as the induction of unfolded protein response and the non-canonical initiation of autophagy [8, 14, 15, 18, 35] or the participation of phagophores in the biogenesis of DMVs.

**Fig 6:**
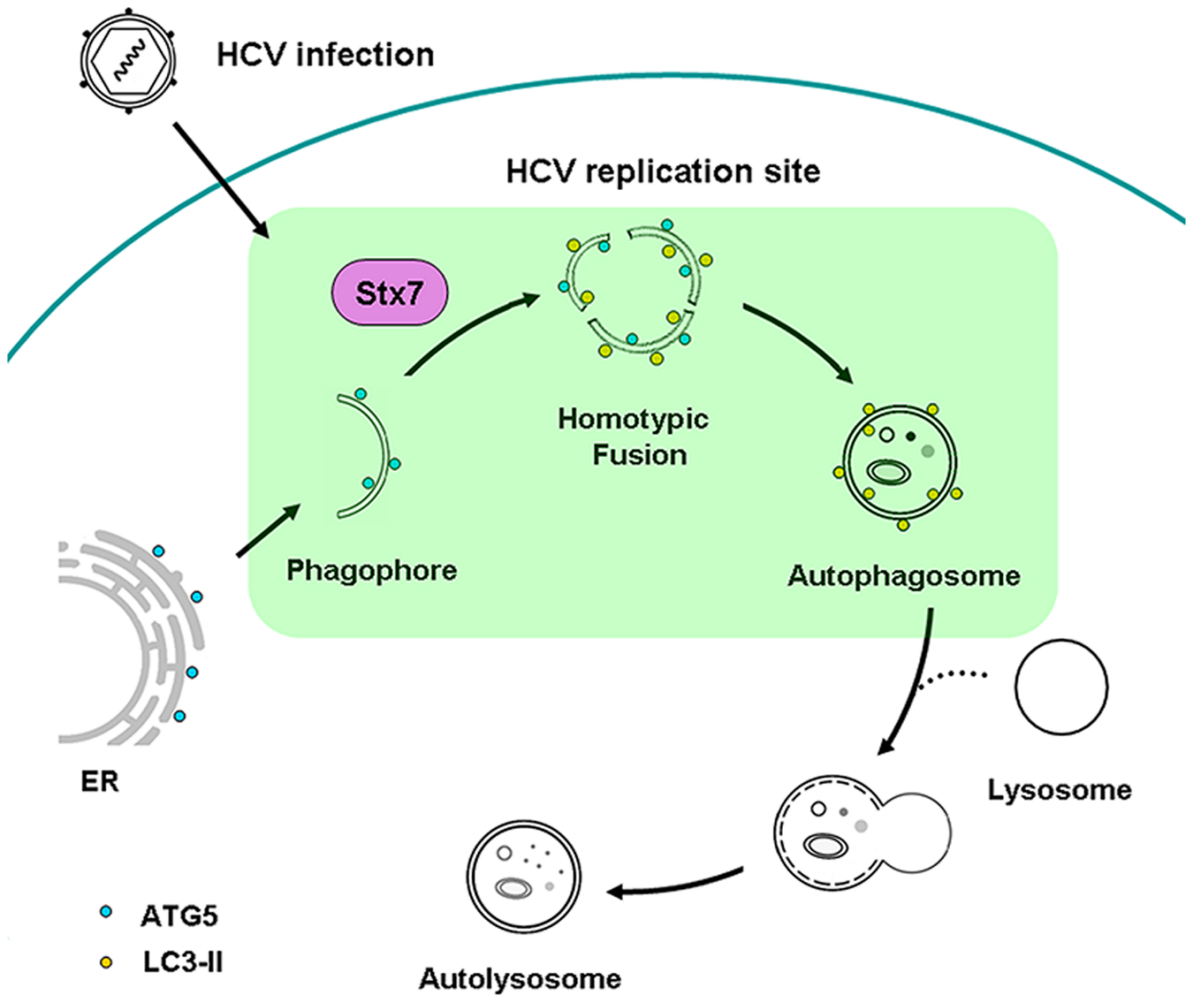
Model for the biogenesis of autophagosomes induced by HCV. HCV infection leads to the localization of ATG5 to the ER and the subsequent formation of phagophores, which undergo homotypic fusion in a STX7-dependent manner to form autophagosomes. Both phagophores and autophagosomes can support HCV RNA replication.

The biogenesis pathway of autophagosomes induced by HCV is summarized in Fig 6. As illustrated in the figure, our results indicated that HCV stimulated the formation of phagophores from the ER, which then underwent homotypic fusion in an STX7-dependent manner to form autophagosomes. We also found that the HCV RNA replication could take place on phagophores, indicating that the HCV RNA replication complex was assembled on autophagosomes in the early stage of its biogenesis and remained associated with autophagosomes after they were generated. It remains to be determined regarding whether phagophores, which contain the HCV RNA replication complex, are also the predecessors of DMVs that had been reported to also mediate HCV RNA replication [38].

## Materials and methods

### Cell cultures

Huh7 and its derivative Huh7.5 (gift of Dr. Charles Rice, Rockefeller University) are human hepatoma cell lines [8]. They were maintained at 37°C in Dulbecco's modified Eagle medium (DMEM) supplemented with 10% fetal bovine serum (FBS) and nonessential amino acids. Huh7N1b replicon cells harboring an HCV subgenomic RNA replicon were maintained in the same medium containing 0.8 mg/ml G418 (Sigma-Aldrich) [39, 40]. Depending on the experiments, cells might be nutrient-starved in Hank’s balanced salt solution (HBSS) for 1 hour. Huh7 cells and HCV replicon cells that stably expressed the GFP-LC3 fusion protein were established by transfecting the cells with the GFP-LC3 expression plasmid followed by selection with hygromycin B (150 μg/ml) and G418 (0.4 mg/ml).

### Antibodies and reagents

The primary antibodies used in this study included the mouse anti-STX7 antibody (Sigma-Aldrich), rabbit anti-ATG5 antibody (Cell Signaling), rabbit anti-p62 antibody (Cell Signaling), mouse anti-HCV NS5A monoclonal antibody (Millipore), rabbit anti-LC3 antibody (Sigma-Aldrich), and rabbit anti-calnexin antibody (Abcam). Proteins were extracted from cell lysates for western-blot analysis using the M-PER mammalian protein extraction reagent (Thermo Fisher Scientific) following the manufacturer’s protocol. The ER Tracker Blue was purchased from Thermo Fisher Scientific.

### Electron microscopy

HCV replicon cells were fixed in 2% glutaraldehyde in neutral phosphate buffer, post-fixed in osmium tetraoxide, and embedded in Epon. Sections were cut at 80 nm and examined under a Philips Tecnai 10 electron microscope.

### Transfection and infection studies

The DNA plasmids mEmerald-ATG5-C-18 (Addgene plasmid #54000) or mCherry-ATG5-C-18 (Addgene plasmid #54995) were mixed with the BioT transfection reagent (Bioland) in serum-free DMEM to a final concentration of 2 μg/mL per the manufacturer’s protocol. This transfection mixture was incubated at room temperature for 20 minutes prior to inoculation into cells. Two days after transfection, cells were harvested for further studies. For infection studies, Huh7.5 cells were infected with HCV using a multiplicity of infection (m.o.i.) of 1 one day after transfection. Infected cells were then harvested one day post-infection for further analysis. All of our infection studies were conducted using a variant of the HCV JFH1 isolate, which replicated more efficiently than the original JFH1 isolate [41].

### *In vitro* membrane fusion assay

The homotypic membrane fusion assay was performed as described [42]. Briefly, two sets of cells were transfected with either the mEmerald-ATG5 or the mCherry-ATG5-expressing plasmid. Cells were harvested in the homogenization buffer (250 mM sucrose, 3 mM imidazole [pH 7.4]) containing the protease inhibitors, and passed through a 25-guage syringe needle 20 times. Cell lysates were centrifuged at 1200xg for 15 min at 4°C. The postnuclear supernatants (PNS) which contained either the mEmerald-ATG5-labeled phagophores or the mCherry-labeled phagophores were mixed with or without an ATP regenerative system for 60 min on ice with shakings in the dark. The mixed cell lysates were then placed on glass slides and fluorescence-labeled phagophores were visualized and imaged using the Keyence All-in-One fluorescence microscope.

### siRNA knockdown of STX7

For the siRNA knockdown experiment, the STX7 siRNA (siSTX7) (SASI_Hs01_00171210) (Sigma-Aldrich) was transfected into cells using Lipofectamine RNAiMAX (Invitrogen) in Opti-MEM (Invitrogen). Briefly, 4 × 10^4^ cells seeded in a 35-mm dish were transfected with 2 μl of siRNA (100 μM each) for 6 hours followed by the replacement of the transfection mixture with fresh DMEM. Replicon cells were harvested 48 hours post-transfection for analysis. For HCV infection studies, one day after the siRNA transfection, Huh7 cells were infected with HCV using an m.o.i. of 1. Cells were then harvested one day after infection for further analysis.

### Purification of phagophores and cell-free HCV RNA replication assay

HCV replicon cell lysates were prepared using previously described procedures with modifications [43]. Briefly, cells grown in 100-mm-diameter dishes were washed with ice-cold phosphate-buffered saline (PBS), followed by treatment with 1-ml per dish ice-cold hypotonic buffer (10 mM Tris-HCl [pH 7.5], 10 mM KCl, 5 mM MgCl2) for 20 minutes. Cells then were scraped off the dish and lysed by passing through a 25-guage syringe needle 20 times. Nuclei and unbroken cells were removed by centrifugation at 1,000xg for 5 min at 4°C. For the membrane-flotation assay, cell lysates were mixed with 3 ml 80% sucrose in low-salt buffer (LSB; 50 mM Tris-HCl [pH 7.5], 25 mM KCl, and 5 mM MgCl2) and overlaid with 4 ml 55% sucrose and then 1.5 ml 10% sucrose in LSB. The sucrose gradient was centrifuged at 38,000 rpm in a Beckman SW40Ti rotor for 14 hours at 4°C. After centrifugation, 1-ml fractions were collected from the top of the gradient. To each fraction, 1.7 ml LSB was added to dilute sucrose, and membranes in individual fractions were concentrated by ultrafiltration using the Amicon Ultra 100K filter (Millipore). Proteins in individual fractions were subjected to western-blot analysis using the ECL-plus system (Thermo Fisher Scientific). For the affinity purification of phagophores, the anti-ATG5 antibody was added to the membrane fraction and after the incubation at 4°C with shaking overnight, protein-G-conjugated Dynabeads (Thermo Fisher Scientific, #10007D) were added and the phagophores were purified using the magnetic separator. For the cell-free HCV RNA replication assay, purified phagophores were incubated with the RNA replication buffer (100 mM HEPES [pH 7.4]; 10 mM KCl; 10 mM MgCl_2_; 0.1 mM MnCl_2_; 5 μg/ml actinomycin D, 1 mM [each] ATP, GTP, and UTP; 10 μM CTP; 3 μCi α-^32^P-CTP [3000 Ci/mmol]) for 2 hours at 30°C. The RNA was extracted from the reaction mixture with TRIzol (Invitrogen). After the addition of 10 μg tRNA carrier and CHCl_3_ and the incubation at room temperature for 10 minutes, the samples were centrifuged at 12000xg for 10 minutes. The RNA was then precipitated after the addition of an equal volume of 4 M ammonium acetate and four volumes of 100% ethanol. The precipitated RNA was resuspended in H_2_O, loaded on a 1% agarose gel containing formaldehyde for electrophoresis, and analyzed by autoradiography.

## Supporting information

S1 Fig VAMP7 knockdown suppresses the formation of autophagosomes without affecting phagophores in nutrient starved cells and HCV replicon cells. (A) Huh7 cells and HCV replicon cells that stably expressed the GFP-LC3 fusion protein were transfected with the mCherry-ATG5-expressing plasmid for one day and then further transfected with the control siRNA (siNC) or VAMP7 siRNA (siVAMP7) for two days. For nutrient starvation, Huh7 cells were incubated in HBSS solution for one hour. Cells were then fixed for fluorescence microscopy. (B) Western-blot analysis of control Huh7 cells, nutrient-starved cells and replicon cells with and without VAMP7 knockdown.
(TIF)

S2 Fig Colocalization analysis of endogenous ATG5 and LC3 with the ER. HCV replicon cells or Huh7 cells infected by HCV for one days were fixed and stained for ATG5 (red), LC3 (green) and ER Tracker (blue) and analyzed by immunofluorescence microscopy.
(TIF)

S3 Fig Colocalization analysis of endogenous ATG16 with ectopically expressed mCherry-ATG5 in control Huh7 cells, HCV replicon cells and HCV-infected cells. Huh7 cells and HCV replicon cells were transfected with the mCherry-ATG5-expressing plasmid for two day and stained with ER Tracker blue, then fixed for immunofluorescence microscopy for ATG16 (green). HCV-infected cells that expressed mCherry-ATG5 were also analyzed.
(TIF)

S4 Fig Inhibition of STX7 expression does not affect the appearance of ATG5 puncta on the ER. (A) Cells were transfected with mEmerald-ATG5 for 1 day and then with the control siRNA (siNC) or siSTX7 for 2 days. For nutrient starvation, cells were starved for one hour, and for HCV infection, one day after siRNA transfection, cells were infected with HCV for one more day. Cells were then fixed and stained for the ER using the anti-calnexin antibody. (B) Percentages of ATG5 puncta colocalized with ER (i.e. Yellow/Green ratio). The results represent the average of >20 cells that were analyzed.
(TIF)

S5 Fig *In vitro* HCV RNA replication assay. Phagophores enriched by the membrane-flotation centrifugation were affinity-purified with either the anti-ATG antibody or the control IgG and used for the HCV RNA replication assay.
(TIF)

S1 Video Live cell imaging of HCV replicon cells. HCV replicon cells that stably expressed LC3-GFP were transfected with the mCherry-ATG5-expressing plasmid for 2 days and then stained with ER Tracker Blue for 40 minutes before imaging. The HCV replicon cells were observed for 40 mins with images taken once every 8 minutes.
(MOV)

S2 Video Live cell imaging of nutrient-starved Huh7 cells. Huh7 cells that stably expressed LC3-GFP were transfected with the mCherry-ATG5-expressing plasmid for 2 days and then stained with ER Tracker Blue for 40 minutes before imaging. Huh7 cells were starved for 1 hour and observed for 10 minutes with images taken once every 2 minutes.
(MOV)
