# Supplementary figures and images for: HCV-induced autophagosomes are generated via homotypic fusion of phagophores that mediate HCV RNA replication
Source: PLoS Pathog. 2017 Sep 19;13(9):e1006609. doi: 10.1371/journal.ppat.1006609 (PMC5621699; doi:10.1371/journal.ppat.1006609)

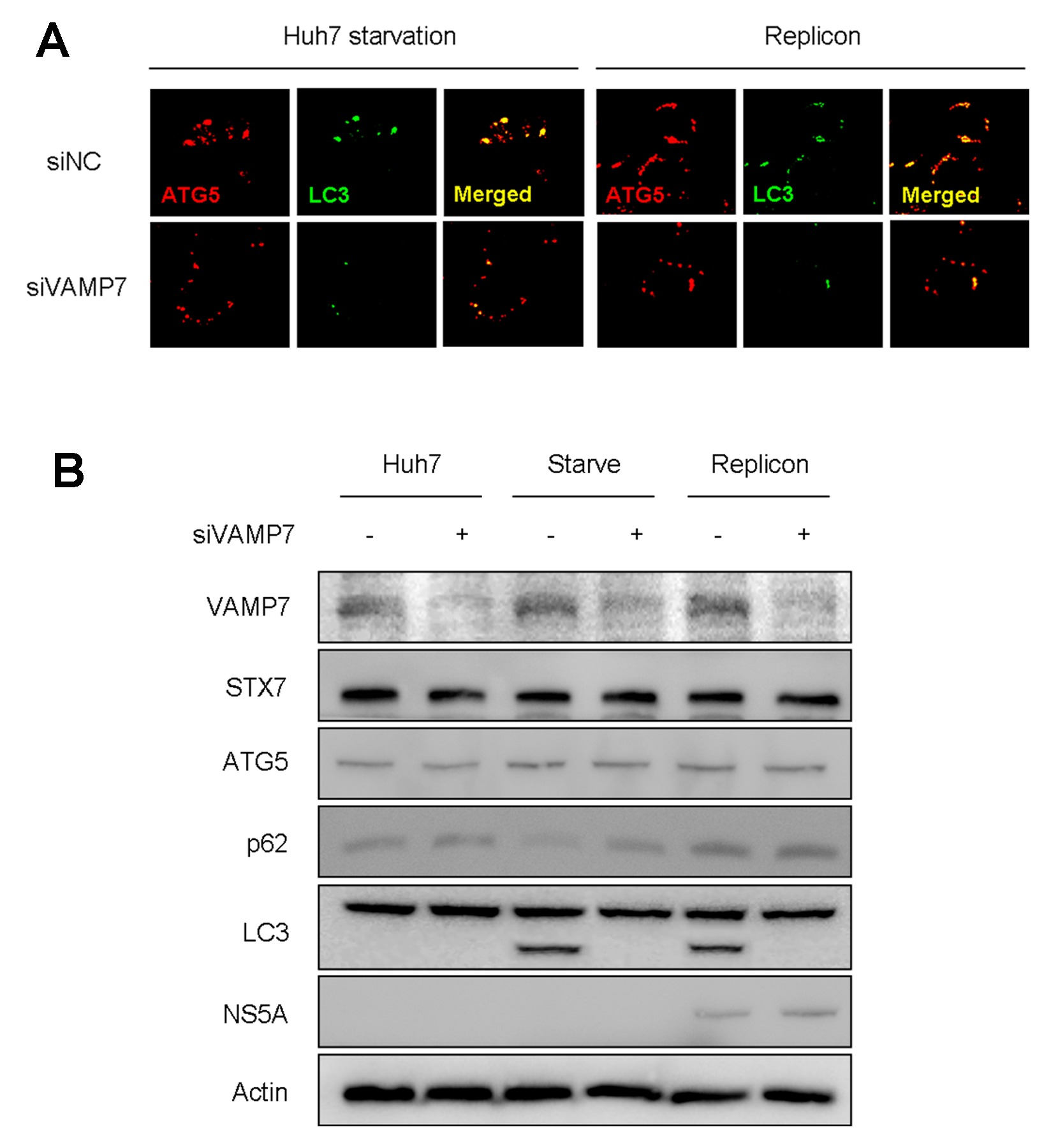

Supplement: S1 Fig — (A) Huh7 cells and HCV replicon cells that stably expressed the GFP-LC3 fusion protein were transfected with the mCherry-ATG5-expressing plasmid for one day and then further transfected with the control siRNA (siNC) or VAMP7 siRNA (siVAMP7) for two days. For nutrient starvation, Huh7 cells were incubated in HBSS solution for one hour. Cells were then fixed for fluorescence microscopy. (B) Western-blot analysis of control Huh7 cells, nutrient-starved cells and replicon cells with and without VAMP7 knockdown. (TIF) [file ppat.1006609.s001.tif]

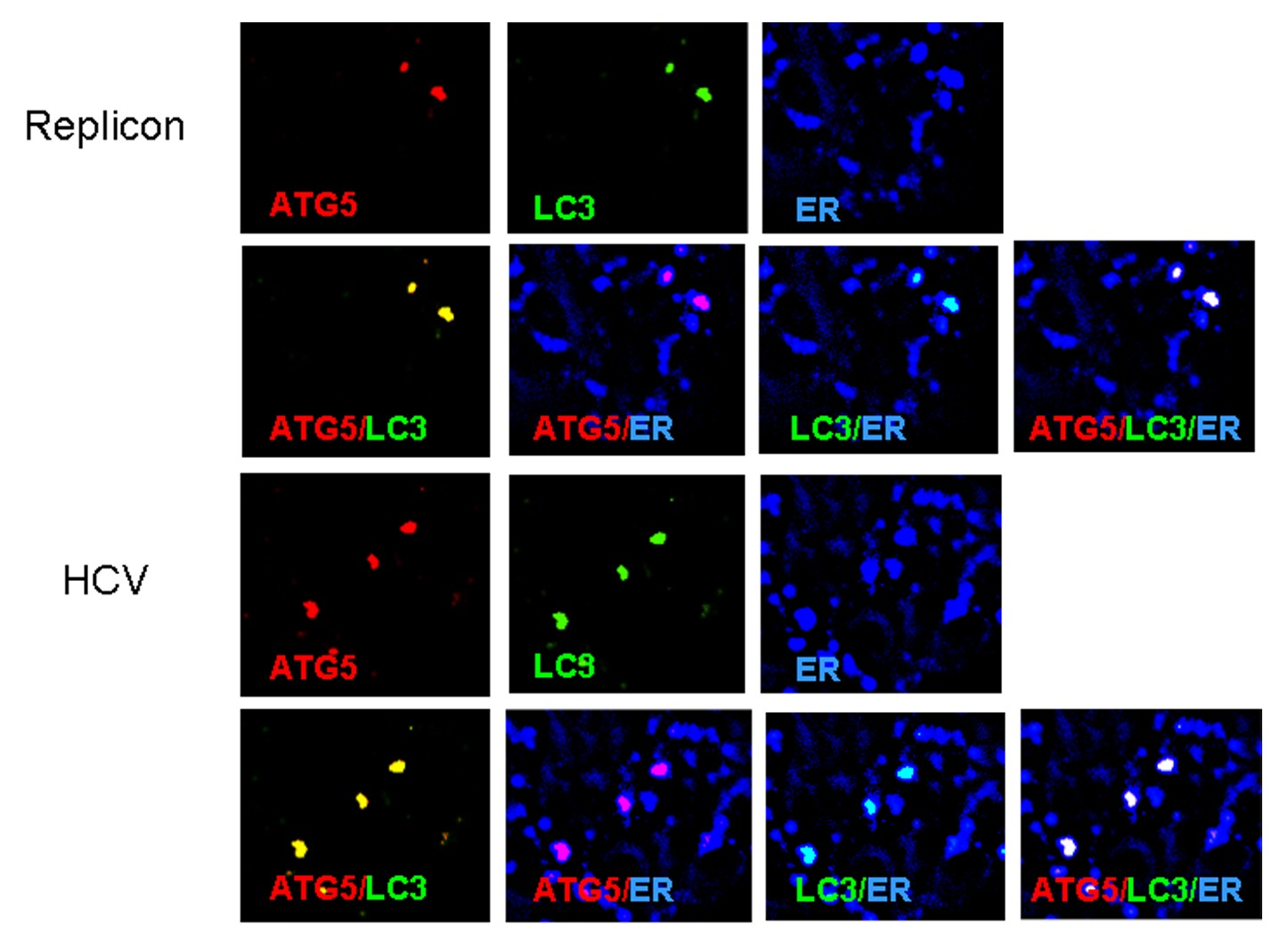

Supplement: S2 Fig — HCV replicon cells or Huh7 cells infected by HCV for one days were fixed and stained for ATG5 (red), LC3 (green) and ER Tracker (blue) and analyzed by immunofluorescence microscopy. (TIF) [file ppat.1006609.s002.tif]

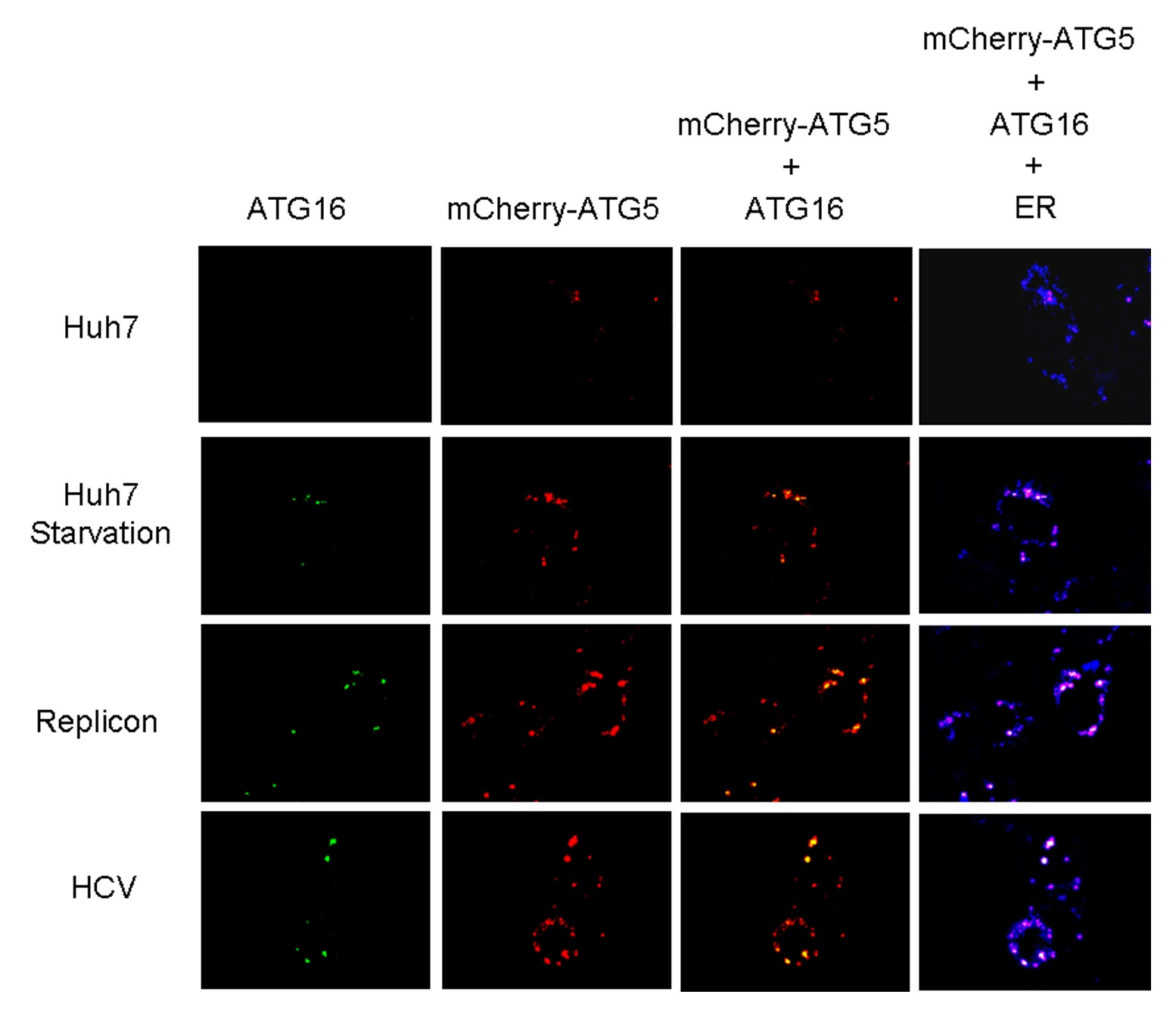

Supplement: S3 Fig — Huh7 cells and HCV replicon cells were transfected with the mCherry-ATG5-expressing plasmid for two day and stained with ER Tracker blue, then fixed for immunofluorescence microscopy for ATG16 (green). HCV-infected cells that expressed mCherry-ATG5 were also analyzed. (TIF) [file ppat.1006609.s003.tif]

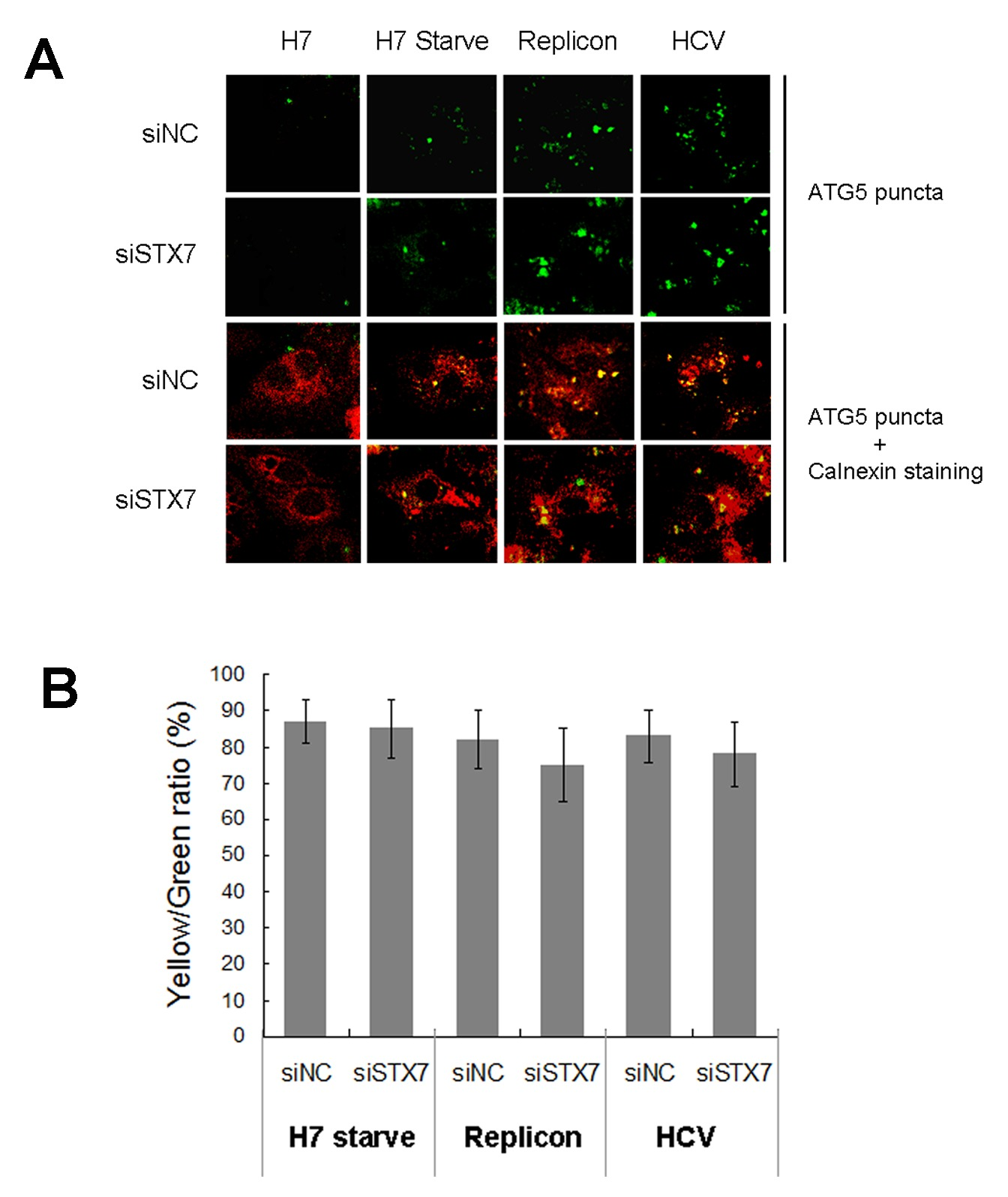

Supplement: S4 Fig — (A) Cells were transfected with mEmerald-ATG5 for 1 day and then with the control siRNA (siNC) or siSTX7 for 2 days. For nutrient starvation, cells were starved for one hour, and for HCV infection, one day after siRNA transfection, cells were infected with HCV for one more day. Cells were then fixed and stained for the ER using the anti-calnexin antibody. (B) Percentages of ATG5 puncta colocalized with ER (i.e. Yellow/Green ratio). The results represent the average of >20 cells that were analyzed. (TIF) [file ppat.1006609.s004.tif]

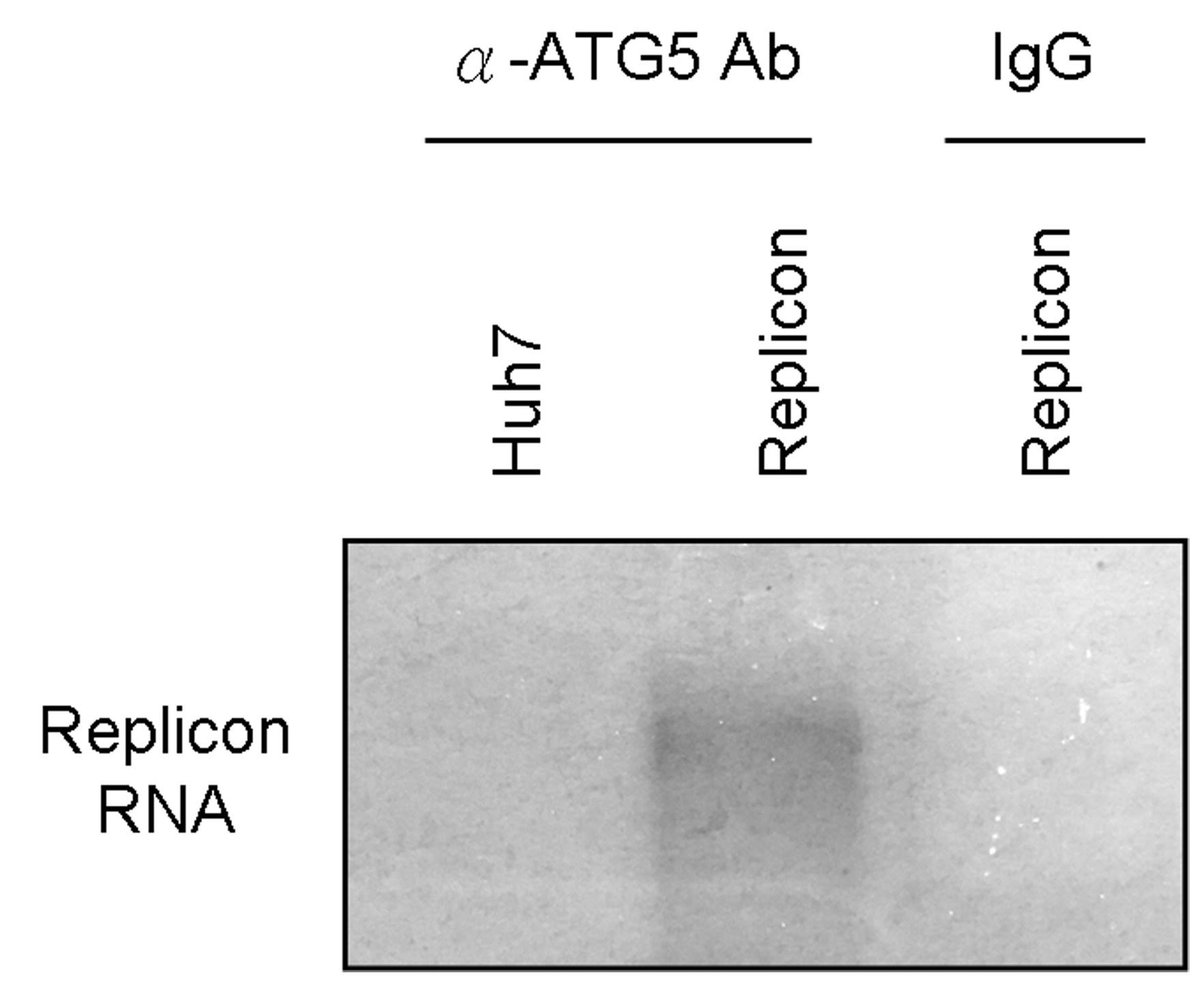

Supplement: S5 Fig — Phagophores enriched by the membrane-flotation centrifugation were affinity-purified with either the anti-ATG antibody or the control IgG and used for the HCV RNA replication assay. (TIF) [file ppat.1006609.s005.tif]
